# Pterins as Diagnostic Markers of Mechanical and Impact-Induced Trauma: A Systematic Review

**DOI:** 10.3390/jcm8091383

**Published:** 2019-09-03

**Authors:** Angus Lindsay, Gregory Baxter-Parker, Steven P. Gieseg

**Affiliations:** 1 Institution for Physical Activity and Nutrition (IPAN), School of Exercise and Nutrition Sciences, Deakin University, Geelong, VIC 3220, Australia; 2 School of Biological Sciences, University of Canterbury, Christchurch 8140, New Zealand; 3 Department of Radiology, University of Otago Christchurch, P.O. Box 4345, Christchurch 8011, New Zealand

**Keywords:** biopterin, exercise, neopterin, surgery, tetrahydrobiopterin, trauma, traumatic brain injury

## Abstract

We performed a systematic review of the literature to evaluate pterins as biomarkers of mechanical and impact-induced trauma. MEDLINE and Scopus were searched in March 2019. We included in vivo human studies that measured a pterin in response to mechanical or impact-induced trauma with no underlying prior disease or complication. We included 40 studies with a total of 3829 subjects. Seventy-seven percent of studies measured a significant increase in a pterin, primarily neopterin or total neopterin (neopterin + 7,8-dihydroneopterin). Fifty-one percent of studies measured an increase within 24 h of trauma, while 46% measured increases beyond 48 h. Pterins also showed promise as predictors of post-trauma complications such as sepsis, multi-organ failure and mortality. Exercise-induced trauma and traumatic brain injury caused an immediate increase in neopterin or total neopterin, while patients of multiple trauma had elevated pterin levels that remained above baseline for several days. Pterin concentration changes in response to surgery were variable with patients undergoing cardiac surgery having immediate and sustained pterin increases, while gastrectomy, liver resection or hysterectomy showed no change. This review provides systematic evidence that pterins, in particular neopterin and total neopterin, increase in response to multiple forms of mechanical or impact-induced trauma.

## 1. Introduction

Pterins are biologically active bi-cyclic compounds. Pterins have been utilised as biomarkers because of their involvement in inflammation [1], oxidative stress [2] and aromatic amino acid hydroxylation [3] since they were discovered in 1889 [4]. Neopterin and tetrahydrobiopterin (BH_4_) are examples of pterins used to assess changes in immune system activation and oxidative status, as well as deficiencies in monoamine neurotransmitter synthesis and nitric oxide availability, respectively. Neopterin is the oxidative product of 7,8-dihydroneopterin, a metabolic derivative of guanosine triphosphate (GTP) produced by monocytes and macrophages during γ-interferon (γ-IFN)-stimulated immune activation [5]. Neopterin has been used a useful measure of exercise-induced stress [6], cancer [7], neuromuscular disease [8], human immunodeficiency virus (HIV) [9], sepsis [10] and vascular disease [11]. BH_4_ is also a product of GTP metabolism and used to diagnose numerous neurological diseases, including phenylketonuria, due to its involvement as a co-factor in many hydroxylase enzyme reactions [12].

Accurate diagnosis and management of mechanical or impact-induced trauma is critical for patient monitoring and treatment. Major mechanical incident can cause immediate structural damage that results in the loss of muscle plasmalemma integrity, organ perforation or bone breaks and a secondary insult of immune cells [13]. Next, a co-ordinated inflammatory and anti-inflammatory response [14] work to remove and remodel damaged tissue while also maintaining homeostasis. In severe cases, infection, systemic inflammatory response syndrome (SIRS), sepsis, multi-organ dysfunction (MOD) and even mortality can occur [15,16,17]. The critical junction between patient admittance and initial treatment can determine the level of morbidity and mortality [18]. Thus, the need for sensitive and accurate diagnostic and prognostic biomarkers of trauma is critical for initial diagnosis, monitoring of patient progress and treatment efficacy.

Pterins are also useful biomarkers of mechanical and impact-induced trauma. Since 1988 [19], studies have utilised the biomarker potential of pterins to accurately assess the extent of initial trauma and the subsequent monitoring of injury progression or recovery. In this systematic review, we sought to gather all published literature measuring pterins in response to mechanical or impact-induced trauma in humans with the purpose of understanding if pterins provide a sensitive and accurate biomarker.

## 2. Experimental Section

*Systematic Review:* We performed a thorough systematic search of the research literature for the use of pterins as biomarkers of mechanical and impact-induced trauma. We performed the literature search in MEDLINE and Scopus on 7 March 2019. The search terms were “pterin” OR “dihydropterin” OR “neopterin” OR “dihydroneopterin” OR “biopterin” OR “dihydrobiopterin” OR “tetrahydrobiopterin” OR “dihydroxanthopterin” OR “xanthopterin” OR “isoxanthopterin” OR “sepiapterin” OR “monapterin” OR “oncopterin” OR “leucopterin” AND “muscle damage” OR “trauma” OR “accident” OR “exercise” OR “sport” OR “surgery”. We delimited studies to those in English and involving human subjects.

*Study Inclusion and Exclusion Criteria:* We considered studies for review that met the following inclusion criteria: (1) a pterin was measured, (2) severe mechanical or impact-induced trauma was evident and (3) the study was conducted on humans. We included exercise that had a significant impact component that resulted in muscle damage, as well as studies that were accidental (car accident) or elective (surgery). We included studies that measured pterins in all media, including urine, plasma, serum, cerebrospinal fluid (CSF), saliva or tissue and did not exclude studies based on design, unless they were systematic or scoping reviews and meta-analyses. Studies meeting the following exclusion criteria were not considered for review: (1) patients undergoing transplant surgery, (2) pterin supplementation studies, (3) surgery where an underlying disease existed unless accompanied by select co-morbidities such as hypertension of diabetes (specified in the surgical table), (4) sepsis unless resulting from trauma, (5) studies on animals, (6) ex vivo pterin analysis of tissue collected from patients following trauma or (7) burn injuries. Finally, there were no exclusion criteria for race, ethnicity, age, sex, nationality, socioeconomic status or education level of the subjects. After the initial systematic review, all authors appropriately screened the articles and disagreements were resolved through discussion. A meta-analysis was not completed because reporting of absolute pterin concentrations were sparse between studies.

## 3. Results

**Figure 1:**
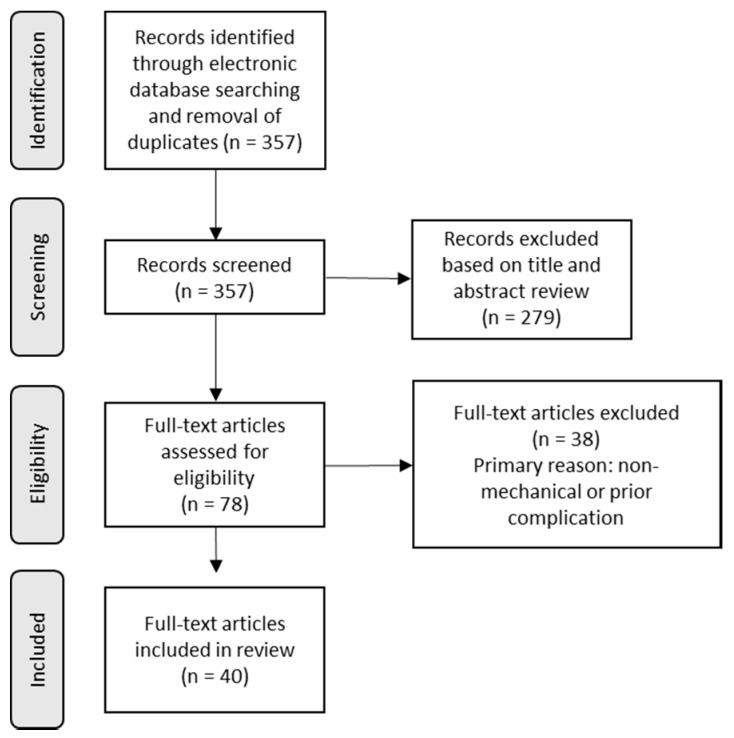
A flow diagram detailing the inclusion/exclusion identification of studies in this systematic review.

*Selection of Studies:* We identified 357 publications through the electronic databases search after removal of duplicates. After review of the title and abstract, we excluded 279 articles. We fully evaluated 78 full-text articles. We excluded 37 articles based on the inclusion/exclusion criteria, resulting in 40 total articles for the systematic review (Figure 1). We separated included articles into four categories of mechanical or impact-induced trauma; exercise/sport, surgery, traumatic brain injury (TBI) and multiple injuries (polytrauma). We detailed individual study results in Table 1, Table 2, Table 3 and Table 4, including inclusion/exclusion criteria and the primary outcome of the pterin response. Therefore, we will not present the detailed results of each included study, but rather an overview of the collective findings.

**Figure 2:**
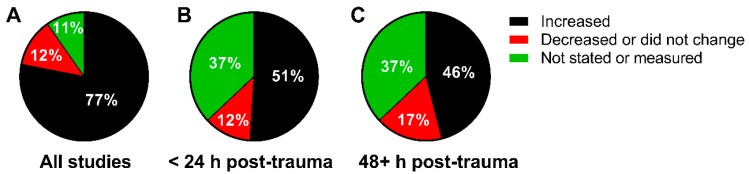
(**A**) Percent of studies that measured an increase, decrease or no change, or did not state or measure changes in pterins. (**B**) Studies that reported a change in pterin concentration within 24 hours of trauma or (**C**) greater than 48 hours post-trauma.

*Overall Finding:* A total of 3829 subjects from 40 studies have been compiled (Figure 2). Seventy-seven percent of the studies reported an increase in a pterin in response to mechanical or impact-induced trauma (Figure 2A). In the studies that reported a pterin response timeline to trauma, 51% increased within 24 hours (Figure 2B), while 46% remained elevated for greater than 48 hours (Figure 2C).

*Exercise Trauma:* Nine total articles met the inclusion criteria for exercise-induced trauma (Table 1). Articles were published between 2015–2017, indicating the use of pterins in exercise-induced trauma is relatively novel. All nine articles measured total neopterin (neopterin + 7,8-dihydroneopterin), six measured neopterin and total neopterin and one measured biopterin, total biopterin (biopterin + 7,8-dihydrobiopterin + BH_4_) and xanthopterin. Each study included elite amateur or professional athletes and measured a change in urinary pterins associated with rugby union or mixed martial arts (MMA), two sports that both endure repeated high-force impacts resulting in muscle damage and inflammation [20]. The evidence indicates a single game of rugby union or an MMA contest or training session causes an immediate increase in all pterins measured. It is noted that an increase in pterins may be specific to the impacts of the sport because neopterin concentrations do not always change in response to exercise devoid of impacts [21]. Changes in neopterin are transient following rugby union, returning to baseline within 24 h of a game while it can remain elevated following an MMA training session up to 24 h post-contest. Interestingly, only a continuous increase in neopterin and total neopterin showed evidence of sustained oxidative stress/inflammation over an extended training camp in MMA (6 weeks), while a whole season of rugby union did not affect concentrations, indicating the intensity and repetition of impact during MMA training may be of a greater intensity than rugby union. Collectively, the data indicates that pterins, in particular neopterin and total neopterin, are sensitive markers of exercise/sport-induced trauma and reflect changes in oxidative stress and inflammation.

*Surgery:* Fifteen articles met the inclusion criteria for surgery-induced trauma (1988–2019; Table 2). Pterins were measured primarily following cardiac surgery with or without cardiopulmonary bypass, but were also measured following herniotomy, cholecystectomy, rectal or colon surgery, ypsilon graft, liver resection, hernia, vulvectomy or hysterectomy, gastrectomy or knee replacement. All 15 articles measured either neopterin or total neopterin, while only one measured BH_4_, indicating a preference for a marker of immune system activation. Furthermore, all but one study measured these pterins in either plasma or serum. All but two articles also had specific exclusion criteria to evaluate the change in biomarker concentrations to the surgical procedure, including pterins (detailed in Table 2). One study investigated the change in neopterin in neonates and infants/children, while all other studies recruited adults. Procedures that involved a form of cardiac surgery always resulted in a significant increase in neopterin within 6-hours post-surgery that remained elevated for several days. Neopterin was also higher in those patients that developed post-surgical complications such as sepsis, delirium, cardiac dysfunction or acute kidney injury following surgery. A similar neopterin response was also observed in patients undergoing hernia surgery or knee replacement surgery; however, there were limited changes in neopterin in response to all other surgeries while some patients showed a decrease in concentration post-surgery, likely because of pre-surgical complications associated with immune system activation or interactions with general anaesthesia down-regulating the immune system. Taken together, the evidence indicates neopterin may only increase in response to cardiac surgery and other limited surgical procedures and identify patients at greater risk of post-surgical complications.

*Traumatic Brain Injury:* Two articles met the inclusion criteria for pterin changes in response to TBI ([44,45]; Table 3). Both articles, published in 2001 and 2002, were by the same group and measured neopterin concentrations in patients in response to an “isolated” or “severe” TBI. Patients presented with a Glasgow Coma Score at admission equal to or less than eight and associated abnormalities of the brain via computer-aided tomography. Neopterin was measured in serum and CSF. In one study, serum neopterin increased in response to the TBI and remained elevated 14 days post-surgery. Neopterin in CSF also increased and remained elevated up to 21 days post-surgery. The second study only presented CSF neopterin concentrations in relation to another biomarker, but some patients had levels > 30 ng/mL. No further studies have measured any pterin in response to TBI since 2002. The limited data available from these two studies does indicate TBI increases plasma neopterin concentrations.

*Multiple Injuries (Polytrauma):* Sixteen total articles published between 1989 and 2017 met the inclusion criteria for multiple injury-induced trauma on changes in pterins (Table 4). All studies involved severe trauma or hip-fracture and patients were greater than 16 years of age. In severe trauma studies, patients were included based on their injury severity score (≥ 16 to a maximum of 66 in a single case-study) or APACHE (acute physiology, age, chronic health evaluation) score (severity of disease; 18.9). Exclusion criteria, where stated, included patients with an underlying disease, immunosuppressive therapy, immunodeficiences, corticosteroid treatment or in some cases, patients did not survive during the data collection period. All 16 studies measured neopterin with only one measuring total neopterin. No studies measured other pterins. Neopterin and total neopterin were measured in urine, serum, plasma and CSF. In all studies, neopterin and total neopterin significantly increased. Neopterin concentrations remained elevated 2–14 days post-trauma, which corresponded with the final measurement of a study design. Neopterin concentrations, irrespective of the bio-fluid, correlated with the severity of trauma [(ISS (injury severity score) and APACHE score] and were higher in non-surviving patients, patients suffering from organ failure as a result of trauma (one study did not see a relationship), patients with delirium and/or cognitive impairment. Neopterin also continually increases in the days following trauma and is a good predictor of mortality up to one-year post-bone fracture in the elderly. Overall, multiple trauma or bone fracture causes an increase in neopterin concentrations that are associated with clinically relevant outcomes, including death.

## 4. Discussion

The purpose of this systematic review was to critically evaluate studies that measured pterins in response to mechanical or impact-induced trauma. Only studies measuring a pterin in humans in response to mechanical or impact-induced trauma were considered and it is the first survey to date of pterins as diagnostic and prognostic biomarkers of trauma. Overall, this review provides evidence that pterins increase in response to trauma. However, different effects and changes in pterin concentration were identified when the type of trauma was considered. Collectively, pterins, particularly neopterin and total neopterin, increase after trauma, remain elevated and may be a predictor of several clinically important patient outcomes.

Review of the literature identified 40 articles that measured pterins in response to trauma, all of which measured either neopterin, total neopterin or both. 7,8-Dihydroneopterin is an antioxidant synthesized from γ-IFN-activated monocytes/macrophages during immune system activation and can be oxidized to neopterin. Neopterin’s highly fluorescent properties and easy detection methods [6,61,62] make it a popular candidate to measure an inflammatory response, which may explain why every article considered in this review measured it in reaction to various forms of trauma. However, the use of neopterin and not the simultaneous measurement of 7,8-dihydroneopteirn as a marker of immune system activation has been a topic of discussion. It is suggested that the measurement of neopterin alone might only be an indicator of oxidative stress and thus, does not reflect the true level of inflammation [2,63]. Therefore, the increase in neopterin associated with mechanical or impact-induced trauma might be a reflection of elevated oxidative stress rather than cumulative inflammation. However, the simultaneous increase in neopterin and total neopterin following knee replacement surgery [43], an MMA contest [27,29,30] or rugby game [22] suggests neopterin might reflect immune system activation in response to trauma as well as oxidative stress.

While neopterin was measured in every study, biopterin, BH_4_ and total biopterin were also measured in response to trauma. BH_4_ is enzymatically synthesized from GTP and metabolized to dihydrobiopterin while acting as a co-factor for the amino acid hydroxylases and nitric oxide synthases. Dihydrobiopterin is salvaged to BH_4_ or oxidized to biopterin. Thus, the measurement of biopterins can be indicative of oxidative stress, monoamine neurotransmitter synthesis and nitric oxide availability (nitric oxide-induced vasodilation and other functions [64]). Given the biopterins ability to indicate several acute and chronic biological conditions, and trauma is associated with oxidative stress and blood flow [65,66,67], it was surprising the biopterins have not been measured in abundance. The lack of biopterins as biomarkers may be due to the multitude of processes that produce biopterin, or use BH_4_ as a co-factor. The only article that clearly states a change in biopterins noted significant increases following four rugby games that was positively associated with the number of impacts a player experienced during the game [28]. Therefore, we suggest, on limited data, that biopterins may provide a general but sensitive biomarker of trauma and should be considered in future studies. Furthermore, xanthopterin was the only other pterin measured, once again in the same study on several rugby games. Xanthopterin, a catabolite of biopterin, was also increased following a rugby game and associated with the number of impacts experienced by a player. Collectively, this evidence and other research showing isoxanthopterin is elevated in muscle degenerative diseases [68], indicates other pterins may be useful indicators of trauma.

Pterins are synthesized from all cell types and tissues. Therefore, measurement of pterins in response to mechanical or impact-induced trauma can be taken in multiple bio-fluids with similar efficacy. While pterin measurements were limited to urine and CSF in exercise and brain injury studies, respectively, pterins were measured almost selectively in plasma or serum in multi-trauma or surgical studies. Blood and CSF media do provide a reliable and direct measurement of pterins in circulation. However, urinary pterins also increase in response to various trauma and it does provide a medium that can be readily accessed for multiple measurements through catheterization or non-invasively using a collection container. Urinary pterins are also able to be measured by HPLC, RIA or ELISA and require less sample clean up prior to measurement; however, urinary pterin concentrations require normalization by creatinine, specific gravity or osmolality to account for changes in hydration. Collectively, pterins increase in all bio-fluids following mechanical or impact-induced trauma, but for longitudinal analyses that require follow-up measurements, urine may be a suitable media.

*Exercise Trauma:* Studies on exercise-induced trauma, typically those associated with repeated high-force impacts like rugby or MMA, have utilized biomarkers like creatine kinase [69], myoglobin [70] or C-reactive protein [71] to measure changes in muscle damage and inflammation. The results of this review provide evidence that repeated high-force impacts in rugby union or MMA always result in a transient increase in several pterins and that this increase is mostly associated with the number of total impacts experienced by a competitor. Furthermore, the studies that collected samples at multiple time-points following exercise noted that neopterin or total neopterin was sustained for up to 24 hours, indicating they can be used to monitor post-traumatic changes in immune system activation following impact-induced trauma [30]. Two studies further defined the potential of total neopterin to measure the longitudinal response to repetitive insult, which might mimic a multiple-trauma patient undergoing repeated surgical intervention and long hospital stays. During a 20-week rugby season, where players complete several training sessions (fitness and weight-lifting) and a competitive game on a weekly basis, total neopterin levels did not change [25]. However, during a six-week MMA training camp where athletes complete weight-lifting and several contact sessions/week, neopterin and total neopterin steadily increased throughout the duration [29]. When assessing exercise-induced trauma using pterins, the evidence suggests pterins should be immediately quantified to understand the acute stress, while weekly monitoring may provide important information about adaptation to repeated insults or development of over-training. Overall, the evidence indicates neopterin and total neopterin are sensitive biomarkers of impact-induced trauma in sport and exercise.

*Surgery:* Surgical intervention is highly invasive and initiates a cascade of inflammatory processes [72]. The results of this review indicate neopterin and/or total neopterin increase with selective surgeries. Therefore, neopterin and/or total neopterin is a useful diagnostic and prognostic marker of surgical intervention. Interestingly, the pterins were only responsive to selective surgeries, which suggests they may only be useful under specific circumstances. For example, any thoracic surgery, with or without cardiopulmonary bypass, always resulted in an increase [35], which likely reflects the level of trauma inflicted on the patient to access the heart. It would be interesting to compare neopterin levels following cardiac key hole surgery. Surgeries such as hysterectomy [39] or gastrectomy [42] did not affect neopterin concentrations, suggesting there is a much lower level of trauma involved in these particular procedures. Differentiating between the level of stress associated with each surgical procedure is difficult but the rise in neopterin is not thoracic-specific, with knee replacement surgery [43] also resulting in an increase. There is also evidence that neopterin remains elevated for several days post-surgery [37] and is higher in patients that develop sepsis [31] and become delirious [38]. Therefore, in a clinical setting, the pre to post change in pterins could provide an assessment of acute surgical stress while long-term daily monitoring of pterins might be advantageous for selective patients that develop post-surgical complications. Because neopterin and total neopterin are considered reliable and sensitive markers of oxidative stress and inflammation, clinicians should consider daily measurements until concentrations either return to “normal” values or remain steady below pre-surgical levels for several days. These data suggest neopterin can provide indicative data on the acute and chronic response to selective surgical interventions, especially where post-operative infection complications are suspected.

*Traumatic Brain Injury:* Traumatic brain injury is associated with inflammation [73] and oxidative stress [74]. While limited studies have measured any pterin in response to TBI, two studies which have noted a significant increase in neopterin also reported a sustained elevation for 14–21 days post-surgery [44,45]. Whether the increase in neopterin is a measure of oxidative stress or inflammation is unknown but it provides evidence that neopterin may be a sensitive marker of TBI. However, because surgery followed the TBI, the surgical intervention could have an effect on neopterin concentrations. It is also interesting that no further studies have explored the use of neopterin or any other pterin in response to TBI given the sensitivity of the two published studies. While numerous studies are exploring biomarkers of TBI [75], perhaps the inclusion of neopterin and/or total neopterin may provide some added diagnostic and prognostic benefit. Therefore, in cases of TBI that arise from sport, accidents or work-related injuries, neopterin and/or total neopterin should be measured at patient admittance and daily until values return to “normal”.

*Multiple Trauma (Polytrauma):* Multiple trauma always results in an acute and chronic increase in neopterin and total neopterin, the latter only being measured in one study [59]. The sensitivity of neopterin in response to multiple trauma seems greatest compared to the three other categories of this review. Neopterin concentrations, whether in the urine, plasma, blood or CSF, significantly increase, but because 15 of the 16 studies only measured neopterin and not total neopterin, the full scope of neopterin’s diagnostic and prognostic capability is unclear. Nonetheless, it is evident that multiple trauma [54] or a bone fracture [60] results in a sustained increase in neopterin. Most importantly, it is the association of neopterin with clinically relevant outcomes like organ failure [50], survival [49] and the severity of the trauma [53] that indicate neopterin is a strong biomarker candidate for identifying the degree of multiple trauma and the likelihood of developing post-trauma complications. Therefore, in cases where patients experience multi-trauma, the evidence suggests neopterin and/or total neopterin should be immediately assessed to quantify the extent of injury and then repeatedly measured every 12–24 hours to monitor development of other complications. Because neopterin was always greater in non-surviving patients, it may provide sensitive and reliable information to clinicians regarding an appropriate course of treatment.

*Limitations:* There are limitations to this systematic review: (1) the number of studies measuring a pterin following traumatic brain injury is severely limited. It is difficult to assess the effectiveness of pterins as biomarkers of TBI, specifically neopterin, in their clinical capacity to monitor the severity and progression of injury. (2) The purpose of this review was to evaluate the response of all pterins to mechanical or impact-induced trauma, yet the current literature is severely limited to neopterin studies. Because other pterins like isoxanthopterin, sepiapterin, pterin and xanthopterin have been measured in diseases like cancer [76] and Duchenne muscular dystrophy [68], and these diseases are associated with inflammation, oxidative stress and ischemia, these pterins may also provide some benefit in situations of trauma. Furthermore, the development of novel nanotechnology/biomedical engineering platforms for biomarker discovery in conditions associated with inflammation and oxidative stress [77,78,79] could further enhance the validity and utility of specialist diagnostic biomarkers such as pterins. (3) It was evident that the media used to measure a selected pterin both within and between each of the four categories was different. For example, all exercise-based studies used urine, whereas all surgical studies used plasma or serum. To understand the capacity of pterins to act as diagnostic and prognostic markers, all media should be considered for measurement of pterins.

## 5. Conclusions

This systematic review evaluated 40 articles that measured pterins in response to mechanical or impact-induced trauma. While neopterin and/or total neopterin were the most quantified pterin, the majority of studies in each of the four categories showed a transient and/or chronic increase. The evidence suggests neopterin and/or total neopterin provide a quality assessment of oxidative stress and inflammation in response to mechanical or impact-induced trauma. Further research on the response of other pterins to mechanical or impact-induced trauma is required before drawing conclusions.

## Figures and Tables

**Table 1:** Studies measuring pterins following impact-induced trauma in exercise and sport.

Authors	Year	Pterin	Media	Population	Intervention	Outcome
Lindsay et al. [22]	2015	NP and Total NP	Urine corrected with specific gravity	Elite amateur rugby players; n = 11; median height = 1.87 m; median weight = 96 kg	Rugby game	- NP ↑ (p = 0.02) - Total NP ↑ (p = 0.008) - Both ↑ within 60 min post-game and retuned to baseline within 17 h and remained until 86 h
Lindsay et al. [23]	2015	Total NP	Urine corrected with specific gravity	Professional rugby players; n = 37; height = 1.81– 2.01 m; weight = 92.9– 112.3 kg; age = 23.7–27.5 years	Several rugby games separated by player position	↑ (43.6–109.6%) within 60 min post-game
Lindsay et al. [24]	2015	Total NP	Urine corrected with specific gravity	Professional rugby players; n = 37; height = 1.86 ± 0.07 m; weight = 104.5 ± 9.3 kg; age = 26 ± 3.5 years	Rugby game – data from several games compiled together during a season	↑ (64%) within 60 min post-game. Still significantly elevated 36 hours post-game
Lindsay et al. [25]	2015	Total NP	Urine corrected with specific gravity	Professional rugby players; n = 37; height = 1.86 ± 0.07 m; weight = 104.5 ± 9.3 kg; age = 26 ± 3.5 years	Rugby season (20 weeks)	No change throughout the season
Lindsay et al. [26]	2015	NP and Total NP	Urine corrected with specific gravity	Semi-professional rugby players; n = 24; height = 1.87 ± 0.06 m; weight = 103.3 ± 11.6 kg; age = 24.2 ± 2.9 years	Three rugby games separated by a week	- NP ↑ (p < 0.002) - Total NP ↑ (1.96–2.41 fold; p ≤ 0.003) - Both positively correlated with number of impacts Both ↑ immediately post-each game
Lindsay et al. [27]	2015	NP and Total NP	Urine corrected with specific gravity	Semi-professional MMA; n = 10; height = 1.77 ± 0.04 m; weight = 79.5 ± 0.5 kg; age = 27.3 ± 3.3 years	MMA fight	- NP ↑ (p = 0.02) 1 h post-fight but was not different up to 48 hours post - Total NP did not change
Lindsay et al. [28]	2016	NP, 7,8-NP, BP, Total BP, XP	Urine corrected with specific gravity	Professional rugby players; n = 14–23; height = 1.86 ± 0.07 m; weight = 104.5 ± 9.3 kg; age = 26 ± 3.5 years	Rugby game—four separate games separated by a week	- NP ↑ (1.37–2.82 fold) - Total NP ↑ (1.45–1.89 fold) - BP ↑ (1.28–1.36 fold) - Total BP ↑ (1.49–1.85 fold) - XP ↑ (0.84–1.57 fold) - All ↑ within 90 min post-game
Lindsay et al. [29]	2016	NP and Total NP	Urine corrected with specific gravity	Semi-professional MMA; n = 14; height = 1.78 ± 0.08 m; weight = 84.3 ± 12.9 kg; age = 26.6 ± 8.2 years	Six-week MMA training camp	NP and Total NP ↑ (p < 0.05–0.001) throughout the 6-week training duration
Lindsay et al. [30]	2017	NP and Total NP	Urine corrected with specific gravity	Semi-professional MMA; n = 15; height = 1.8 ± 0.09 m; weight = 88.8 ± 14.5 kg; age = 28.3 ± 5.7 years	MMA training session	- NP ↑ 4.07 fold and remained elevated up to 24 hours post - Total NP ↑ 3.15 fold and remained elevated 24 hours post

BP; biopterin, MMA; mixed martial arts or artists, NP; Neopterin, Total BP; BP + BH_2_ (7,8-dihydrobiopterin) + BH_4_ (5,6,7,8-tetrahydrobiopterin), Total NP; NP + 7,8-dihydroneopterin, XP; xanthopterin.

**Table 2:** Studies measuring pterins following surgery.

Authors	Year	Pterin	Media	Population	Categorization	Outcome
Grob et al. [19]	1988	NP	Serum	Consecutive patients hospitalised for selective surgery; n = 35/surgery (10 females and 25 males); age 18–72 years	Herniotomy, cholecystectomy, major surgery on rectum or colon, aorto-coronary bypass or ypsilon graft	No change within 4-days post-surgery among all surgery types
Pilz et al. [31]	1994	NP	Plasma	Patients excluding heart transplantation and pacemaker implantation; n = 110; age = 58.4 ± 1.1 (no complications) and 61.0 ± 2.3 (septic complication)	Elective cardiac surgery	- ↑ after day 1 and remained elevated after 3 days - Delayed ↑ after extracorporeal circulation - Greater ↑ in septic complication patients post-surgery
Bogă et al. [32]	2000	NP	Plasma	Patients excluding those undergoing emergency surgery, or having a diagnosed systemic disorder such as hemostatic defect, hypertension, diabetes or renal failure; n = 40 (10 female and 30 male); age = 57.8 ± 8.9 (study group) and 61.2 ± 7.5 years (control group)	Coronary artery bypass grafting—with or without ultrafiltration	- mean NP ↓ after surgery and ↑ 3- and 20-hours post-surgery (statistical analysis not provided) - no difference with ultrafiltration
Jerin et al. [33]	2002	NP	Serum	Patients suffered from malignant disease (hepatic metastases of colonic cancer, hepatocellular cancer, gallbladder cancer or cholangiocarcinoma) or non-malignant disease (echinococcus cysts, liver haemangioma, liver adenoma or Carroli disease); n = 27 (16 female and 11 male); age 5–77 years	Liver resection	- No change 24-hours post-surgery - NP higher in patients with malignant disease.
Schwab et al. [34]	2004	NP	Serum	No exclusions; n = 101; height = 162– 196 cm; weight = 53–108 kg; age = 18–90 years	Hernia surgery—local or general anaesthetic and either unilateral or bilateral repair	NP ↑ significantly 1 h post-surgery but no difference between the surgical groups. NP was still elevated 72 hours post-surgery
Brkić et al. [35]	2006	NP	Serum	Patients excluding those undergoing reoperations, combined procedures or emergency surgery, those with acute or chronic renal failure requiring hemodialysis and known inflammatory diseases requiring antibiotics or steroids; n = 78 (21 female and 57 male); age = 56–68 years	Cardiac surgery with or without cardiopulmonary bypass	- Both surgeries ↑ NP at 24 and 72 h post-surgery with bypass further ↑ concentrations - NP correlated with ICU stay duration
Skrak et al. [36]	2007	NP	Serum	Patients with a congenital heart defect; n = 152; age = 11 days–13 years; weight = 3.3–44 kg	Cardiac surgery with cardiopulmonary bypass	NP ↑ in neonates and infants/children after one day and remained elevated after 2 days
Forrest et al. [37]	2011	NP	Serum	No data on inclusion – control group were major non-thoracic surgery patients; n = 28 (3 female and 25 male); age = 60.2 ± 1.7 years	Cardiac bypass surgery	- Cardiac surgery ↓ NP compared to baseline but ↑ 6 days-post - Thoracic surgery did not affect NP
Osse et al. [38]	2012	Total NP BH_4_	Plasma	Patients excluding those whom required deep cooling, circulatory arrest, or emergency surgery; n = 125 provided pterin samples; median age = 76 years	Cardiac surgery—coronary artery bypass graft or valve surgery	- NP ↑ following surgery. Elevated pre-surgical NP associated with a greater risk of becoming delirious after surgery - BH_4_ did not change or correlate with development of delirium
Hol et al. [39]	2014	Total NP	Plasma	Patients > 18 years of age and ASA classification I-III; n = 28 (all female);age = 62 ± 12 and 44 ± 9 years; height = 166 ± 5 and 167 ± 6 cm; weight = 71 ± 10 and 71 ± 7	Vulvectomy or abdominal hysterectomy	- Surgery ↓ total NP for both surgical groups up to 24 hours post - Vulvectomy patients had higher concentrations pre and post-surgery
Berg et al. [40]	2015	NP	Plasma	Patients excluding those with infectious blood, active endocarditis, underwent off-pump surgery, intercurrent infection or elevated levels of CRP; n = 1018 (282 female and 736 male); age = 67–75 years	Cardiac surgery	Improved the accuracy of predicting cardiac dysfunction after surgery
Enger et al. [41]	2017	NP	Plasma	Patients without preoperative dialysis or missing serum creatinine; n = 1015 (282 female and 733 male);age = 66–76 years;body mass index = 26.6–29.4 kg/m^2^	Cardiac surgery with cardiopulmonary bypass	Independent predictor of CSA-AKI
Christensen et al. [42]	2018	NP	Plasma	Patients had at least one obesity related comorbidity (such as T2D, hypertension or dyslipidaemia); n = 37 (25 females and 12 males);age = 42.5–53.5 years;body mass index = 40.9–47.6	Laparoscopic sleeve gastrectomyor biliopancreatic diversion with duodenal switch	No change up to 12 months post-surgery
Baxter-Parker et al. [43]	2019	NP and Total NP	Urine	Patients were excluded if below 18 or above 80 years of age, smokers, or patients having recently received a diagnosis of cancer.; n = 19; age = 62.68 ± 8.97 years. Control subjects; n = 20; age = 37.7 ± 12.9	Knee replacement surgery	- NP ↑ two days post-surgery - Total NP did not change in surgical group - NP and total NP elevated versus control 2- and 1-days following surgery, respectively

ASA; American Society of Anesthesiologists, BH_4_ (5,6,7,8-tetrahydrobiopterin), CRP; C-reactive protein, CSA-AKI; acute kidney injury following cardiac surgery, NP; Neopterin, T2D; type 2 diabetes, Total BP; BP + BH_2_ (7,8-dihydrobiopterin) + BH_4_ (5,6,7,8-tetrahydrobiopterin), Total NP; NP + 7,8-dihydroneopterin.

**Table 3:** Studies measuring pterins following traumatic brain injury.

Authors	Year	Pterin	Media	Population	Categorization	Outcome
Lenzlinger et al. [44]	2001	NP	CSF and Serum	Patients presented with a Glasgow Coma Score at admission equal to or less than 8 and/or abnormalities in the computer aided tomography of the brain; n = 41 (9 female and 32 male); age 38 ± 17 years	Isolated TBI	- CSF NP ↑ in 78% of patients and remained elevated 21 days post-surgery (1.88–39.48 ng/mL) - Serum NP ↑ in 73% of patients and remained elevated 14 days post-surgery (0.59–40.05 ng/mL)
Lenzlinger et al. [45]	2002	NP	CSF	Patients had a Glasgow Coma Scale (GCS) score equal to or less than 8 at admission and alterations in the computer aided tomography; n = 10 (4 female and 6 male); age 18–65 years	Severe TBI	No directly stated values but examination of the results suggest values ranged from approximately 0–40 ng/mL

CSF; cerebrospinal fluid, NP; Neopterin, TBI; traumatic brain injury.

**Table 4:** Studies measuring pterins following multiple trauma or bone fracture.

Authors	Year	Pterin	Media	Population	Categorization	Outcome
Brandl et al. [46]	1989	NP	Serum	Injures were initially quantified according to the ISS; n = 51 but NP measured in 26	Multiple-trauma	NP higher in non-survivors
Krüger et al. [47]	1991	NP	Serum	Patients had no acute disease process prior to trauma. All patients had multiple fractures of the extremities, blunt trauma, and a hypovolaemic shock. The Glasgow coma score was above 8 points within 6 h post trauma n = 16; age 36 ± 17 years	Polytrauma	NP ↑ after trauma (values not provided)
Strohmaier et al. [48]	1992	NP	Plasma	Case study—38-year-old female with ISS of 66	Trauma-accident victim—leg amputation	NP ↑ during the clinical course
Waydhas et al. [49]	1992	NP	Plasma	Patients admitted to the emergency department with less than 6 hours between accident and admission to the emergency department; between 16 and 70 years of age; and severe injuries of at least two body regions (head/brain, thorax, abdomen, skeletal system) or three major fractures (ISS ≥ 32; n = 100 (36 female and 74 male); age = 38 years	Trauma	- NP ↑ within 24 h - NP higher in non-survivors from 2 days post-admission - NP higher in surviving patients with organ failure
Roumen et al. [50]	1995	NP	Urine (creatinine standardized)	Patients were included in the study if the ISS was ≥ 33; n = 56	Multiple trauma	NP higher in patients with multiple organ failure once it had become established 8–10 days post-trauma
Hobisch-Hagen et al. [51]	2001	NP	Serum	Patients aged >19 yrs, expected, stay in the ICU > 72 h Partial pressure of oxygen (PaO_2_) > 75 torr during the ICU stay, no renal or hepatic failure, no history of hematopoietic or endocrinological disorder, and ISS ≥ 30; n = 23 (5 female and 18 male); age = 19–59 years	Multiple mechanical trauma	NP low after admission but ↑ until 9 days post-trauma
Hensler et al. [52]	2003	NP	Plasma	Patients were ≥ 16 years of age, ISS ≥ 16 and an expected minimum of survival for ≥ 3 days. Patients with acquired or inherited immunodeficiencies and patients receiving immunosuppressive therapy were excluded; n = 137 (35 female and 102 male); age = 38.5 ± 16.6 years	Trauma, including brain injury	- NP ↓ post-trauma and ↑ 1- and 2-days post - NP not a predictor of patients developing sepsis or multi-organ failure
Egger et al. [53]	2004	NP	Blood – not specified	Patients who died or who were transported abroad where the further course could not be followed were excluded from the study. Eight patients with an ISS of ≤ 20, 10 patients with an ISS of 21–30, and eight patients with an ISS > 30 were included; n = 26 (8 female and 18 male); age = 17–77 years;	Multiple trauma	- NP ↑ in some patients (unclear at what time point) - NP correlated with the severity of trauma
Walsh et al. [54]	2005	NP	Urine (creatinine standardized)	Patients aged 18 to 50 with an ISS > 20 were included. Patients that received corticosteroids for head injuries or died within 1 week from severe mechanical injury were excluded	Severe injury	- mean NP ↑ 1-day post-trauma - NP ↑ in the 13 days post-injury and was higher in patients developing sepsis
Ploder et al. [55]	2006	NP	Plasma	Patients were aged between 18 and 80 years and multiple trauma, defined as injury to two or more anatomic areas (head, chest, abdomen, or pelvis) or to one anatomic area and to two long bones (femur, tibia, or humerus). Exclusion criteria were isolated head injury, known immunosuppressive therapy, or known HIV infection; n = 21; age = 38.7 ± 15.8 years. Mean ISS = 40.6 ± 11.6	Multiple trauma	NP continually increased above entry to ICU for 14 days and was higher in non-survivors
Ploder et al. [56]	2008	NP	Serum	Patients with an APACHE score of 18.9 ± 6.75 and ISS score of 39 ± 13.1; n = 18 (4 female and 14 male); age = 45 ± 19 years.	Trauma	- NP ↑ in patients with 79.8% above the 95^th^ percentile of healthy controls - NP and APACHE scores correlate 4-days post-trauma
Ploder et al. [57]	2009	NP	Serum	Patients aged between 22 - 77 years and evidence of a multiple trauma (ISS = 39.1 ± 13.1; APACHE = 17.5 ± 6.5). Exclusion criteria were known immunosuppressive therapy, known HIV infection or any other chronic disease; n = 15 (3 female and 12 male); age = 40.4 ± 17.2 years	Trauma	- NP ↑ compared to controls - NP greater in patients who died during follow-up (7–37-days post-trauma)
Ploder et al. [58]	2010	NP	Plasma	Patients admitted to ICU. APACHE score: 18.9 ± 6.75, ISS: 39 ± 13.1; n = 18 (4 female and 14 male); age = 45 ± 19 years	Trauma	↑ NP compared to control subjects but timeline could not be determined
Hall et al. [59]	2016	Total NP	CSF and serum	Patients included if the fracture was caused by a low energy trauma (defined as a fall from less than 1 meter). Patients were excluded if they were under the age of 60, were nursing home residents, had significant Parkinson’s disease or had malignant or other comorbid disease such that prognosis was less than one year; n = 139	Hip fracture	- NP values above upper end of normal - NP highest in patients with delirium and/or cognitive impairment
Larsen et al. [60]	2017	NP	Plasma	Patients aged 75 years and older, free of medication and diseases affecting the immune system (e.g., cancers, autoimmune disorders), absence of prior physical disabilities and, absence of cognitive disorders; n = 60	Hip fracture followed by surgery	Predictor of mortality one-year post-fracture and correlates negatively with time of survival after fracture surgery

CRP; C-reactive protein, CSF; cerebrospinal fluid, HIV; human-immunodeficiency virus, ICU; intensive care unit, ISS; injury severity score, NP; Neopterin, T2D; type 2 diabetes, Total NP; NP + 7,8-dihydroneopterin.
